# Acetylcholine in Brain–Body Communication: Biological Mechanisms and Physiological Roles

**DOI:** 10.3390/ijms27114686

**Published:** 2026-05-22

**Authors:** Yuan Gao, Tian Zhou, Xinsheng Lai, Erkang Fei

**Affiliations:** 1 HuanKui Academy, Jiangxi Medical College, Nanchang University, Nanchang 330031, China; 18720061862@163.com (Y.G.); zhoutian@ncu.edu.cn (T.Z.); 2 School of Basic Medical Sciences, Jiangxi Medical College, Nanchang University, Nanchang 330031, China; laixinsheng@ncu.edu.cn; 3 Institute of Biomedical Innovation, Jiangxi Medical College, Nanchang University, Nanchang 330031, China

**Keywords:** acetylcholine, brain–body axis, cholinergic signaling, CNS, PNS

## Abstract

Acetylcholine (ACh) is an evolutionarily conserved neurotransmitter that is widely distributed in the central and peripheral nervous systems and plays essential roles in multiple physiological processes. This review summarizes the full biological cycle of ACh, including its synthesis, vesicular storage, release, degradation, and reuptake, and discusses the regulatory mechanisms underlying its functions in the nervous system and peripheral organs. Through nicotinic acetylcholine receptors (nAChRs) and muscarinic acetylcholine receptors (mAChRs), ACh is involved in central nervous system functions such as cognition, learning and memory, attention, arousal, reward, and decision-making, as well as peripheral processes including motor control, autonomic regulation, and immune modulation. In addition, ACh plays a pivotal role in the brain–body axis. At the central level, the nervous system regulates peripheral organ function through autonomic and neuroendocrine pathways. At the peripheral level, cholinergic signals derived from the enteric nervous system and immune cells convey information about the body’s internal state to the central nervous system through vagal and other afferent pathways, forming an important bottom-up regulatory network. Collectively, these findings indicate that ACh is not only a classical neurotransmitter but also a key molecular mediator of brain–body communication. A more comprehensive understanding of cholinergic signaling may provide new insights into physiological regulation and the pathogenesis of neurological, psychiatric, cardiovascular, and inflammatory diseases.

## 1. Introduction

Acetylcholine (ACh) is one of the most evolutionarily conserved neurotransmitters and is widely distributed throughout both the central and peripheral nervous systems of vertebrates and invertebrates. As a fundamental signaling molecule, ACh exerts diverse physiological functions across multiple organ systems. In addition to regulating complex processes within the central nervous system, it serves as a critical messenger in body–brain communication, particularly in the neuro-immune axis, the autonomic nervous system, and the gut–brain axis [1]. A comprehensive understanding of ACh signaling is therefore essential for elucidating the fundamental mechanisms underlying physiological regulation as well as the initiation and progression of diseases.

The discovery of ACh emerged from early 20th-century investigations into neural transmission. In 1921, Otto Loewi provided the first direct evidence of chemical communication between nerves and target organs through his landmark experiment on vagal stimulation in the frog heart [2]. He demonstrated that stimulation of the vagus nerve released a chemical substance, termed “vagus nerve substance”, which slowed heart rate [3]. Subsequent studies by Henry Dale and colleagues led to the chemical identification of this substance as ACh [4,5]. This discovery represented a major milestone in neuroscience, establishing ACh as the first identified neurotransmitter and fundamentally reshaping the understanding of neuronal communication by demonstrating that synaptic signaling occurs not only through electrical impulses but also through chemical mediators.

This review summarizes the biochemical life cycle of ACh, including its synthesis, storage, release, degradation, and reuptake, and discusses its roles in the body–brain axis from the perspectives of both the central and peripheral nervous systems. ACh plays a pivotal position in bidirectional body–brain communication, modulating diverse physiological functions through multiple neural and non-neural pathways.

## 2. The Biochemical Process of ACh in the Body

**Figure 1:**
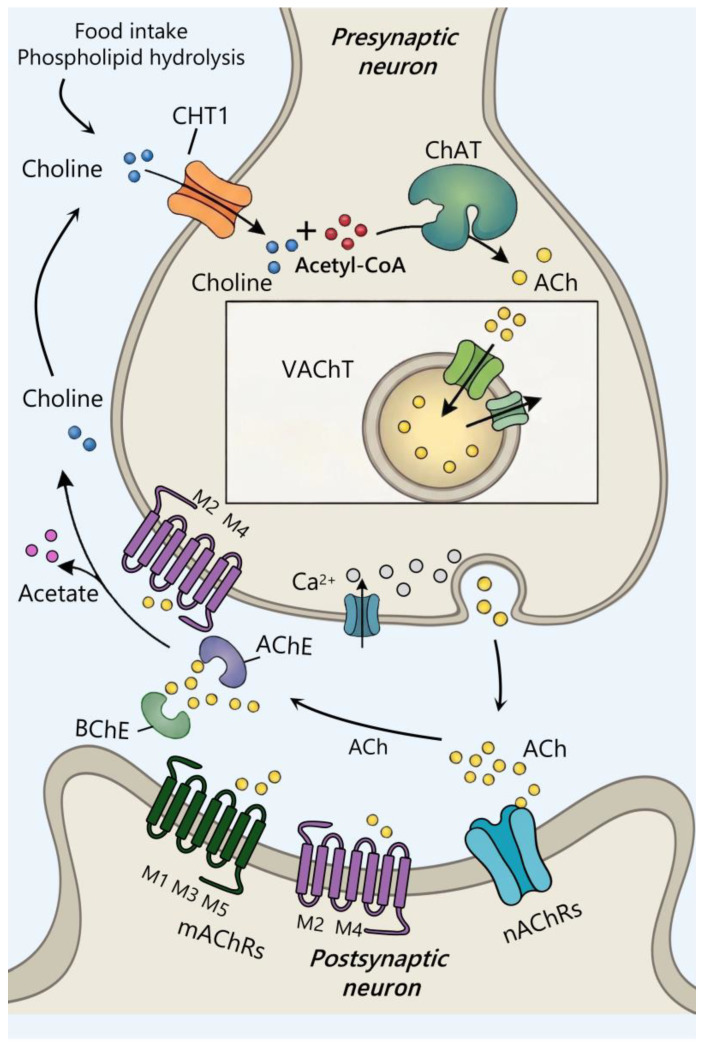
The acetylcholine (ACh) life cycle. ACh is synthesized in the cytoplasm of cholinergic neurons from choline and acetyl-coenzyme (Acetyl-CoA) by the enzyme choline acetyltransferase (ChAT). Choline is taken up from the extracellular space via the high-affinity choline transporter (CHT1). Newly synthesized ACh is transported into synaptic vesicles by the vesicular acetylcholine transporter (VAChT), utilizing the proton electrochemical gradient. Upon arrival of an action potential, voltage-gated calcium channels open, allowing Ca^2+^ influx, which triggers vesicle fusion with the presynaptic membrane and exocytotic release of ACh into the synaptic cleft. In the cleft, ACh binds to nicotinic (nAChRs) and muscarinic (mAChRs) receptors on the postsynaptic membrane to mediate synaptic transmission. The action of ACh is rapidly terminated by hydrolysis into choline and acetate by acetylcholinesterase (AChE) and, to a lesser extent, by butyrylcholinesterase (BChE). The resulting choline is recycled back into the presynaptic neuron via CHT1 for reuse in ACh synthesis.

The ACh life cycle includes biosynthesis, vesicular transport and storage, synaptic release, and degradation with subsequent choline recycling (Figure 1). Genetic ablation or reduced expression of these molecules in mouse models results in a range of phenotypic changes (Table 1), highlighting their indispensable roles in cholinergic signaling. Accordingly, components of the ACh metabolic and transport machinery represent important targets for understanding the pathophysiology of ACh-related disorders and for developing therapeutic interventions.

### 2.1. Synthesis

Cholinergic neurons synthesize ACh in the cytoplasm through a reaction that requires two precursors: choline and acetyl-coenzyme A (acetyl-CoA) [6]. This reaction is catalyzed by choline acetyltransferase (ChAT). Choline is derived from dietary intake, phospholipid hydrolysis, and circulating free choline, and is transported into cholinergic neurons from the extracellular space by the sodium-dependent high-affinity choline transporter (CHT1) (Figure 1) [7]. Because this transport step depends on the sodium gradient [8], it has traditionally been considered the rate-limiting step in the ACh synthesis. However, some studies have suggested that ChAT activity, rather than choline uptake, may be the primary determinant of ACh biosynthesis, as ChAT expression and activity increased markedly after nerve growth factor (NGF) treatment [9]. Acetyl-CoA is mainly generated in mitochondrial glycolysis and the tricarboxylic acid cycle (TCA) [10]. ChAT catalyzes the transfer of an acetyl group from acetyl-CoA to the choline, thereby producing ACh [11].

Genetic studies have highlighted the essential role of ChAT in neuromuscular and neuronal function. *ChAT* knockout mice die at birth and exhibit flaccid paralysis due to complete blockade of neuromuscular transmission [12]. In addition, conditional deletion of *ChAT* in approximately 40% of spinal cord and brainstem motor neurons permits postnatal survive but leads to progressive motor dysfunction and reduced muscle strength after 6 months of age [13]. Selective *ChAT* knockout in rostral brainstem cholinergic nuclei results in markedly decreased ultrasonic vocalization, enhanced sensorimotor gating, and reduced overall activity [14]. Collectively, these findings indicate that abnormalities in ChAT-mediated ACh synthesis are associated with impairments in motor function, muscle development, and social behavior.

### 2.2. Transport and Storage

Following synthesis, ACh is transported into synaptic vesicles by the vesicular acetylcholine transporter (VAChT) [15]. VAChT utilizes the vesicular proton electrochemical gradient to exchange two intravesicular protons for one cytoplasmic ACh molecule [16]. Each vesicle contains thousands of ACh molecules, allowing rapid and efficient neurotransmitter release in response to stimulation (Figure 1). As a major vesicular membrane protein in cholinergic neurons, VAChT is indispensable for cholinergic transmission.

VAChT is a key protein in the synaptic vesicle membrane of cholinergic neurons. Conditional knockout of *VAChT* in mice causes death within 2–5 min after birth, and unreleased newly synthesized ACh can be detected in the brain [17]. *VAChT*-knockdown mice with approximately 70% reduction in expression exhibit cardiac remodeling, impaired calcium handling, and contractile dysfunction, phenotypes reminiscent of early heart failure [18,19]. In the neuromuscular junction (NMJ), VAChT deficiency leads to abnormal synaptic vesicle morphology and reduced transmission efficiency [20]. Moreover, selective deletion or knockdown of *VAChT* in cholinergic interneurons impairs behavioral flexibility, increases habituation, and promotes vulnerability to maladaptive feeding behaviors [21,22]. *VAChT* knockdown has also been associated with increased susceptibility to epilepsy, earlier onset of brain death, increased awakenings during intermediate sleep stages, and impaired episodic memory [23,24]. These findings suggest that VAChT-dependent vesicular storage of ACh is critical for normal NMJ, autonomic, and cognitive functions.

### 2.3. Release

ACh release is a calcium-dependent process of synaptic transmission. When an action potential reaches the presynaptic terminal, voltage-gated calcium channels open and trigger influx of calcium ions [25]. The resulting transient rise in intracellular calcium is the primary signal for vesicle fusion and neurotransmitter release (Figure 1) [26]. Studies have shown that ACh release can only be detected when Ca^2+^ is present outside the cell. This indicates that ACh release is a strictly calcium-dependent process [27]. Calcium ions facilitate fusion between synaptic vesicles and the presynaptic membrane by activating the SNARE protein complex, thereby promoting exocytosis [28,29].

After release into the synaptic cleft, ACh binds to cholinergic receptors on the postsynaptic membrane and induces downstream biological responses. These receptors are primarily divided into nicotinic acetylcholine receptors (nAChRs) and muscarinic acetylcholine receptors (mAChRs) (Figure 1). nAChRs are ligand-gated ion channels comprising α (α1–α10), β (β1–β4), and other subunits arranged as pentamers [30]. The α7 subtype is closely associated with learning and memory, whereas α4β2 receptors are important in nicotine and alcohol addiction [31]. nAChRs are widely expressed at the NMJ as well as in the central and peripheral nervous systems (CNS and PNS), where they mediate rapid synaptic transmission by regulating multiple ion currents [32]. mAChRs belong to the G protein-coupled receptor (GPCR) superfamily and comprise five subtypes, M1-M5 [33]. M1, M3, and M5 are primarily coupled to Gαq proteins and activate phospholipase C (PLC), leading to intracellular calcium mobilization and protein kinase C (PKC) activation [34]. In contrast, M2 and M4 couple to Gαi proteins, inhibit adenylate cyclase via, reduce cyclic AMP (cAMP) levels, and regulate membrane potential and potassium channels activity [35]. mAChRs are widely distributed in the hippocampus, cortex, basal ganglia, and autonomic effector organs such as smooth muscle, cardiac muscle, and glands [36,37,38]. They play important roles in cognition and in the regulation of autonomic function.

### 2.4. Degradation and Recovery of Choline

The action of ACh in the synaptic cleft is brief. It is rapidly hydrolyzed by acetylcholinesterase (AChE) into choline and acetate, thereby terminating synaptic transmission [39]. In addition to AChE, butyrylcholinesterase (BChE) can also hydrolyze ACh (Figure 1). BChE is a serine hydrolase widely distributed in plasma, liver, and brain [40]. When AChE activity is reduced or absent, BChE can partially compensate for ACh hydrolysis [41]. In late-stage Alzheimer’s disease (AD), AChE activity declines markedly, while BChE expression and activity increase, making BChE an attractive therapeutic target [42]. Although BChE has a broader substrate spectrum and can hydrolyze esters such as butyrylcholine and propylcholine, its catalytic efficiency toward ACh is much lower than that of AChE [43].

AChE displays highly specific tissue and site distribution, with the highest activity at the NMJ and in CNS synapses [44,45]. AChE is synthesized in the endoplasmic reticulum (ER) and undergoes glycosylation and folding in the Golgi apparatus [46].

Assembled AChE oligomers must be anchored to the cell membrane to function, and two primary anchoring mechanisms exist for this process. The basal lamina of the NMJ synaptic cleft is an extracellular matrix (ECM) structure located between the nerve terminal and the muscle cell membrane. Its unique ColQ component is one of the main anchoring partners of acetylcholinesterase (AChE). AChE binds to the C-terminal domain of ColQ, and Asymmetric 12 (A12) type of AChE is anchored on the basal membrane of the neuromuscular junction. This anchoring mechanism ensures efficient local hydrolysis of acetylcholine by AChE within the synaptic cleft [47]. Perlecan, an important basement membrane glycoprotein, provides structural support and binds multiple bioactive molecules, including fibroblast growth factor (FGF) and vascular endothelial growth factor (VEGF), thereby contributing to local microenvironmental regulation and synaptic plasticity. In perlecan homozygous knockout mice, AChE is absent from the NMJ, indicating that perlecan is essential for AChE localization at this site [48]. A second anchoring mechanism involves the formation of tetramers (G4) through interaction with proline-rich membrane-anchor (PRiMA), a transmembrane protein that anchors AChE to the cell membrane, including the presynaptic membrane. Membrane-bound AChE can also be released into the synaptic cleft through proteolytic shedding, a process regulated by metalloproteinase and presynaptic muscarinic receptors [49].

**Table 1:** Phenotypic effects of knockout or knockdown of ACh-related genes in mice.

Gene	Manipulation	Target Region	Phenotypic Consequences	Reference
*ChAT*	Knockout	Whole body	Perinatal lethality; complete NMJ blockade	[12]
*ChAT*	Knockout	Spinal cord and brainstem	Progressive decline in motor function and muscle strength over 6 months	[13]
*ChAT*	Knockout	Brainstem	Reduced ultrasonic vocalization and locomotor activity	[14]
*VAChT*	Knockout	Whole body	Death within 2–5 min after birth	[17]
*VAChT*	Knockdown	Myocardium	Impaired cardiac function and cardiac remodeling	[18,19]
*VAChT*	Knockdown	Whole body	Abnormal synaptic vesicle morphology and reduced number at NMJs	[20]
*VAChT*	Knockout	Dorsomedial striatum	Reduced behavioral flexibility and increased habit formation	[21,22]
*VAChT*	Knockdown	Whole body	Increased susceptibility to epilepsy	[23]
*VAChT*	Knockdown	Whole body	Increased awakenings during the middle stage of sleep; impaired episodic memory	[24]
*AChE*	Knockout	Whole body (heterozygous)	No obvious abnormalities	[49]
*AChE*	Knockout	Whole body (homozygous)	Embryonic lethality; Developmental defects	[50]

Earlier studies suggested that loss of AChE function would be lethal. However, experiments in embryonic stem cells demonstrated mice carrying a single functional *AChE* allele can survival and develop normally [50]. Subsequent generation of *AChE*-null mice revealed developmental defects, premature death, and hypersensitivity to cholinesterase inhibitors [51]. These studies refined the understanding of the lethal mechanism of organophosphorus toxicity, indicating that AChE deficiency does not prevent organismal formation but leads to severe functional impairment [52]. Although this phenotype was initially attributed to compensatory upregulation of BChE, Li and colleagues found no significant change in BChE activity in the knockout strain [53]. It has therefore been proposed that adaptive mechanisms during development may allow survival in the absence of AChE [54,55].

After ACh degradation, choline is taken up into presynaptic neurons by the high-affinity choline transporter (CHT1), where it is reused for the synthesis of a new round of ACh [56]. CHT1 actively absorbs choline and exhibits sensitivity to choline concentration. This sodium-dependent recycling process forms an efficient choline-ACh cycle that is essential for sustaining cholinergic neurotransmission [57].

## 3. Functions of ACh in the Brain and Body

Cholinergic neurons are extensive and highly organized throughout the nervous system. Their complex projection patterns underlie the pivotal role of ACh in regulating diverse physiological functions, including autonomic activity, cognition, the sleep–wake cycle, and sensory information processing. These neurons are present both within the central nervous system (CNS) and widely distributed throughout the peripheral nervous system (PNS).

### 3.1. CNS

#### 3.1.1. Distribution of Cholinergic Neurons in the CNS

Within the CNS, ACh functions as a neurotransmitter released by distinct populations of cholinergic neurons, which include long-projecting cholinergic neurons and local cholinergic interneurons [58]. Cholinergic neurons are generally classified into three major types: motor neurons, interneurons, and projection neurons. Cholinergic motor neurons are distributed throughout the hindbrain and spinal cord, whereas cholinergic interneurons are found in the striatal complex and in certain cortical and hippocampal regions, depending on the species. Cholinergic projection neurons are divided into two major pools, one located in the brainstem and the other in the basal forebrain [59]. The basal forebrain is home to the largest population of cholinergic neurons in the brain [60].

Basal forebrain cholinergic neurons are classically divided into four cell groups: Ch1, located in the medial septal nucleus; Ch2, located in the vertical limb of the diagonal band of Broca; Ch3, located in the horizontal limb of the diagonal band of Broca; and Ch4, located in the nucleus basalis of Meynert [61]. Among these, Ch4 represents the largest neuronal group and projects extensively to the cerebral cortex and amygdala [62]. The brainstem cholinergic system includes the pedunculopontine tegmental (PPT) nucleus and the lateral dorsal tegmental (LDT) nucleus [63]. The pedunculopontine nucleus (PPN) is also an important cholinergic region. Cholinergic neurons are relatively sparse in the rostral parietal portion of the PPN, but are more abundant in the caudal paralemniscal region [64].

Cholinergic neurons are also prominently distributed in the striatum, particularly in the caudate nucleus and putamen, where cholinergic marker density is among the highest in the brain [65]. This distribution highlights the crucial role of cholinergic interneurons in striatal function, although their precise mechanisms remain incompletely understood [66]. The cerebellum is another important region receiving cholinergic input. It not only receives extrinsic cholinergic projections but also contains intrinsic cholinergic neurons [67]. Cholinergic fibers innervate nearly the entire cerebellum and are especially abundant in lobules IX and X as well as in other cerebellar lobules [68].

Cholinergic projections to thalamic nuclei, particularly the lateral geniculate nucleus (LGN), have also received considerable attention. In the LGN, cholinergic input enhances the activity of GABAergic interneurons via M2 muscarinic receptors and modulates thalamocortical relay cells through nicotinic and M1 muscarinic receptors [63]. Although cholinergic neurons are relatively sparse in the thalamus, they play an important role in sensory integration and transmission through their extensive connections with the cerebral cortex via the reticular thalamic nucleus.

#### 3.1.2. Functions of ACh in the CNS

##### Cognition, Learning and Memory, Attention and Arousal

Cognitive functions encompass aspects such as memory, learning, attention, and perception. Cholinergic signaling is essential for maintaining normal cognitive performance. Central cholinergic circuits regulate signaling throughout the cerebral cortex and support higher-order cognitive processes [69]. The basal forebrain is the main source of cholinergic output in the CNS [70]. Its projections to the cerebral cortex and hippocampus are critical, and degeneration of these neurons is associated with age-related cognitive decline [71]. Hippocampus is indispensable for learning and memory, and septohippocampal cholinergic pathways are known to contribute to multiple hippocampus-dependent behaviors [72]. Both α7 nAChRs and β2 nAChRs are highly expressed in the hippocampus and participate in diverse cognitive functions [73]. One study found that the integrity of the cholinergic basal forebrain is correlated with the volume of hippocampal subregions [74], and MRI studies have further supported the association between cognitive impairment and cholinergic synaptic loss [75].

Cholinergic neurons also contribute to learning by regulating synaptic plasticity and refining neural signaling [36]. Activation of muscarinic ACh receptors can influence learning by modulating AMPA receptor-mediated synaptic transmission [76]. Because the amygdala is closely involved in learning processes, different cholinergic inputs can convey salient information to the amygdala and thereby shape learning behavior [77]. In addition, Ashley and colleagues reported that striatal cholinergic interneurons modulate skill learning [78].

Memory formation generally involves encoding and consolidation [79]. ACh differentially regulates these two phases and appears to affect spatial rather than procedural memory [80]. Cholinergic signaling is mediated by both metabotropic and ionotropic receptors, with mAChRs functioning as metabotropic receptors and nAChRs as ionotropic receptors [81]. By activating these receptor classes, ACh promotes memory formation and consolidation through enhancement of synaptic plasticity [73]. Memory can be broadly categorized into declarative and non-declarative memory [82]. Declarative memory consolidation depends heavily on medial temporal lobe structures, particularly the hippocampus [83]. Cholinergic inputs from the basal forebrain can induce theta oscillations in the hippocampus and related regions [84], and theta oscillations are thought to support spatial memory encoding [85]. Animal studies have shown that phasic activation of basal forebrain cholinergic neurons enhances the retrieval of already formed memories [86]. In this process, M1 muscarinic receptors on hippocampal pyramidal neurons may play an important role [87].

Attention refers to the selective processing of relevant stimuli while suppressing irrelevant information. As a key neural substrate governing this cognitive function, the cholinergic system regulates attention through both sustained cholinergic modulation and transient cholinergic signaling [88]. The primary anatomical basis of cholinergic modulation lies in the basal forebrain: cholinergic neurons here project broadly to the cortex, particularly the prefrontal cortex, which is crucial for cue detection and top-down control of attention [89]. Beyond the cortex, the hippocampus also receives cholinergic projections from the basal forebrain that contribute to attentional processes [90]. Notably, these basal forebrain cholinergic neurons are influenced by multiple inputs, especially orexinergic neurons from the lateral hypothalamus [91]. Specifically, orexin peptides regulate attention by modulating neuronal activity and ACh release [92]. However, the cholinergic regulation of attention can be disrupted by adverse factors. Stress-induced molecular and morphological changes in cholinergic neurons in the nucleus basalis of Meynert reduce ACh release and impair attentional performance [93]. The regulation of attention is not a single process, but involves multiple interactions that jointly regulate it. In the cortex, attentional regulation is mediated especially through the prefrontal and posterior parietal cortices and their interactions [94]. Recent studies further suggest that cholinergic and dopaminergic neurons interact cooperatively to regulate attention, extending beyond the effects of either system alone [95].

Cholinergic neurons are also crucial for maintaining wakefulness. Basal forebrain cholinergic neurons sustain the waking state by projecting to the cerebral cortex and promoting cortical activation and gamma-band activity [96]. Optogenetic studies have shown that stimulation of basal forebrain cholinergic neurons rapidly induces transitions from slow-wave sleep to wakefulness or REM sleep and promotes arousal [97]. Conversely, inhibition of cholinergic neurons prolongs slow-wave sleep and reduces the likelihood of awakening [98]. These findings indicate that cholinergic neurons are important for both the initiation and maintenance of REM sleep. Cholinergic neurons in the PPT and LDT of the brainstem are considered key regulators of REM sleep [99]. During REM sleep, they activate the thalamus and cortex and promote theta activity in the EEG, thereby contributing to the characteristic features of REM sleep [100]. In addition, cholinergic neurons cooperate with glutamatergic and GABAergic neurons to regulate sleep–wake behavior, influencing arousal and motor activity through distinct axonal projections [101,102].

##### Reward, Motivation, and Decision-Making

Reward is a core mechanism driving behavior and learning. Although the dopaminergic system was long considered the primary mediator of reward processing [103,104], accumulating evidence indicates that ACh also plays a significant role in the reward system. Some effects arise through interactions with dopamine, whereas others reflect direct cholinergic actions. The striatum is typically divided into the dorsal striatum and the ventral striatum, or nucleus accumbens (NAc), which integrates limbic information. Research has shown that the α6 nAChR selectively activates dopaminergic neurons in the striatal midbrain, thereby promoting dopamine release in the striatum [105]. This process influences responses to reward cues and reward-seeking behavior in animals [106]. More recent work has shown that, in addition to direct interactions, spontaneous rhythms of striatal dopamine and ACh can also propagate from outside the striatum [107], offering a new perspective on reward-related behavior.

In the basolateral amygdala (BLA), ACh levels increase markedly during reward-related events. ACh has been proposed as a key upstream neuromodulator of the BLA. Enhancing endogenous cholinergic signaling in the BLA improves mouse performance in cue-learning tasks driven by motivational value [108]. Optogenetic stimulation of cholinergic terminals in the BLA has also been shown to increase time spent in the stimulus-paired chamber, suggesting that ACh release in the BLA may directly contribute to reward-related behavior [77].

Animals assign motivational value, or incentive salience, to cues predicting reward. In the NAc core, cholinergic receptors bidirectionally regulate cue-induced motivational behavior. Blocking mAChRs attenuates cue-induced reward-seeking, whereas blocking nAChRs enhances this motivational effect [109]. The VTA is a key region of the midbrain that is closely associated with cholinergic input. ACh mediates motivational behavior by activating M5 muscarinic receptors in the VTA and regulates dopamine release in the NAc [110]. This suggests that the VTA and NAc can jointly regulate the generation of motivational behavior through their interactions.

Decision-making is the cognitive process of selecting one action from several possible alternatives. The striatum is a key brain region for action selection and reinforcement learning, and its cholinergic interneurons (CINs) play a central role. During decision-making, dopamine and ACh within the striatum display multiphasic and often antagonistic transient dynamics [111]. Dopamine inhibits the activity of CINs via D2 receptors, thereby suppressing ACh release, whereas ACh can promote dopamine release, forming a finely tuned local feedback loop. This reciprocal relationship is important for normal decision-making. In a human study, blockade of M1 receptors increased willingness to exert physical effort for reward, an effect opposite to that of dopamine D2 receptor blockade [112], suggesting that ACh and dopamine may exert antagonistic effects in effort-based decision-making. The role of ventral striatal CINs (vsCINs) also appears to depend on behavioral stage. Recent evidence suggests that during task acquisition, activation of vsCINs reduces risk-taking, whereas inhibition increases risk-taking; this effect may also show sex differences [113]. Once behavior becomes stable, vsCINs appear to regulate motor impulsivity rather than action selection. These findings indicate that the cholinergic system plays distinct roles in the learning and execution phases of decision-making.

### 3.2. Peripheral Nervous System (PNS)

#### 3.2.1. Somatic Nervous System

In the PNS, cholinergic neurons are primarily distributed in the somatic and autonomic nervous system. Motor neurons are a major component of the somatic nervous system. These cholinergic motor neurons are mainly located in the anterior horn of the spinal cord and their axons project to the NMJs of skeletal muscles, where they release ACh to regulate muscle contraction [114]. This constitutes the direct pathway through which voluntary behavior influences peripheral tissue.

The NMJ is a highly specialized chemical synapse between motor neurons and skeletal muscle fibers and includes Schwann cells (SCs), all of which are essential for normal motor function [115]. When a nerve impulse reaches the presynaptic terminal, the influx of calcium ions triggers vesicular release of ACh into the synaptic cleft [116]. ACh then diffuses across the cleft and binds to nAChRs on the postsynaptic muscle membrane, leading to depolarization and generation of an endplate potential (EPP) [117]. When the EPP reaches the threshold, it triggers a muscle action potential, thereby initiating excitation-contraction coupling and muscle contraction [118]. In addition, ACh can also regulate its own release from the presynaptic terminal. Research has shown that ACh inhibits N-type voltage-gated calcium channels by activating presynaptic M2 muscarinic receptors and a nicotinic receptor sensitive to d-tubocurarine, thereby reducing the influx of calcium and inhibiting the release of ACh [119]. This mechanism constitutes an important presynaptic autoregulatory negative feedback loop. It is worth noting that, terminal Schwann cells (TSCs) are a specialized type of glial cell that surround motor nerve terminals and assist them in performing many functions [120]. When ACh accumulates to a sufficient concentration in the synaptic cleft, it activates the α7 nAChRs on TSCs. This prompts TSCs to release gamma-aminobutyric acid (GABA), which in turn activates GABAB receptors on motor nerve terminals, ultimately inhibiting further ACh release [121]. This pathway is particularly active during early development or in congenital myopathy syndromes, and it plays a role in the onset of muscle fatigue.

To ensure accurate signal transmission and timely muscle relaxation, ACh must be rapidly removed from the synaptic cleft. Accordingly, AChE is essential for NMJ function because it terminates continued stimulation of the postsynaptic membrane. AChE deficiency may result in disorders such as congenital myasthenic syndrome (CMS) [115,122]. It is worth highlighting that pyridostigmine and neostigmine are the most commonly used reversible AChE inhibitors in clinical practice, primarily for the treatment of neuromuscular disorders such as myasthenia gravis. These drugs increase the concentration of ACh in the synaptic cleft, thereby enhancing the stability of neuromuscular transmission [123]. Moreover, organophosphorus compounds also warrant special attention. As a major class of irreversible AChE inhibitors, poisoning from organophosphorus compounds leads to excessive accumulation of acetylcholine in the synaptic cleft, resulting in sustained overstimulation of cholinergic receptors and causing a range of clinical syndromes [124]. The development and maintenance of the NMJ depend on numerous proteins within the synaptic basal lamina, which are closely associated with ACh receptor (AChR) subunits and muscle-specific kinase (MuSK), leading to the formation and stabilization of high-density AChR clusters at the NMJ [125]. Nerve terminals also stimulate transcription of AChR subunit genes, thereby enhancing receptor stability and promoting AChR accumulation at the NMJ [126]. Furthermore, cholinergic neurons promote the development and maturation of the neuromuscular junction by releasing neurotrophic factors such as ARIA (Acetylcholine Receptor Inducing Activity Factor) [127]. More broadly, presynaptic and postsynaptic plasticity regulated by cholinergic neurons is crucial for maintaining NMJ function.

Cholinergic neurons also play important roles in motor control, particularly in the basal forebrain and brainstem. Basal forebrain cholinergic neurons regulate the learning and execution of coordinated movements by projecting to the prefrontal and motor cortex [128]. In the primary motor cortex (M1), maturation of motor representations depends on basal forebrain cholinergic inputs [129]. These neurons are especially important during motor skill acquisition [130]. In the brainstem, cholinergic neurons in the PPT and LDT modulate arousal and motor behavior through projections to the thalamus and basal forebrain [131]. In the striatum, cholinergic interneurons integrate voluntary and involuntary actions and participate in motor control [65,132]. Together, these cholinergic systems ensure effective transmission of neural signals to skeletal muscles and contribute to the regulation of movement [132].

#### 3.2.2. Autonomic Nervous System

In the autonomic nervous system, most neurons in the parasympathetic ganglia are cholinergic, with the exception of those in the Edinger–Westphal nucleus. In contrast, cholinergic neurons in the sympathetic system are primarily located in preganglionic fibers [133].

Within the sympathetic nervous system, preganglionic cholinergic fibers control the adrenal medulla and sweat glands. In the parasympathetic nervous system, cholinergic neurons are mainly distributed in the brainstem and sacral spinal cord, where they regulate visceral organs and mediate the “rest and digest” response [134]. The vagus nerve, often described as the body’s major protective neural pathway, consists largely of cholinergic fibers that use ACh as their principal neurotransmitter [135]. Increasing evidence indicates that central mechanisms regulate pro-inflammatory cytokines and other mediators involved in inflammation resolution through efferent vagal cholinergic signaling, with important effects on cardiovascular, gastrointestinal, respiratory, and other physiological processes [136].

##### Sympathetic Nervous System

In the sympathetic nervous system, ACh plays a central role in preganglionic signal transmission. Preganglionic neurons are located in the intermediolateral column of the thoracolumbar region (T1-L2), and their axons project to paravertebral or prevertebral sympathetic ganglia [137]. When an action potential reaches the presynaptic terminal, it triggers vesicular release of ACh [138]. The released ACh then diffuse across the synaptic cleft and binds to nAChR on the postsynaptic neuronal membrane [139], constituting the first step in sympathetic signal transmission.

Notably, nAChRs in sympathetic ganglia show subunit specificity, being composed primarily of α3 and β4 subunits [140,141]. Studies have shown that expression of α3-nAChR in sympathetic ganglia remains stable even after brain death, further highlighting its importance in sympathetic ganglion function [142]. Although the sympathetic nervous system is classically associated with norepinephrine (NE)-mediated “fight-or-flight” responses, ACh may coexist with or complement NE in certain contexts. For example, following myocardial infarction, sympathetic neurons innervating the heart can switch to ACh production, thereby slowing heart rate and reducing contractile force [143]. Further studies using ChAT-deficient sympathetic neurons identified electrophysiological consequences of this sympathetic cholinergic transdifferentiation, providing new insight into ACh/NE co-release in cardiac regulation [144].

The adrenal medulla represents the neuroendocrine branch of the sympathetic nervous system. During stress, preganglionic sympathetic neurons release ACh, which activates nAChRs on chromaffin cells and ultimately induces secretion of catecholamines and vasoactive peptides [145].

##### Parasympathetic Nervous System

As the primary neurotransmitter of the parasympathetic nervous system, ACh participates throughout the entire signaling pathway from neuronal transmission to effector organ responses, forming the neurochemical basis of the “rest and digestion” state [146]. In contrast to postganglionic sympathetic fibers, which primarily release norepinephrine, all postganglionic parasympathetic fibers are cholinergic and release ACh, which acts on mAChRs to regulate bodily functions during relaxation [147].

In most species, including humans, the heart is innervated by the parasympathetic nervous system throughout all regions, although supraventricular tissue receives denser innervation than the ventricles [148]. Vagal ACh primarily acts on M2 receptors in the sinoatrial and atrioventricular nodes [149]. M2 receptors are coupled to inhibitory G proteins (Gi/o). Upon activation, the βγ subunits of these G proteins directly open G protein-coupled inwardly rectifying potassium channels (GIRK). The resulting potassium efflux hyperpolarizes the membrane, making it more difficult for pacemaker cells to reach the action potential threshold, thereby slowing heart rate and atrioventricular conduction [150,151]. This mechanism is essential for maintaining resting heart rate and cardiovascular homeostasis. In addition, microinjection of ACh into the periaqueductal gray matter (PAG) significantly reduces blood pressure and heart rate via muscarinic receptors [152], suggesting that ACh also contributes to fine regulation of blood pressure.

ACh also induces smooth muscle contraction through mAChRs, representing one of the classical functions of the parasympathetic nervous system. In most smooth muscle cells, the mAChRs are predominantly composed of M2 and M3 subtypes at an approximate ratio of 80% to 20% [153]. Traditionally, M3 receptors have been considered the main mediators of contraction. M3 receptors couple to Gq proteins, activate phospholipase C-β (PLC-β), and promote hydrolysis of phosphatidylinositol 4,5-bisphosphate (PIP2) into inositol trisphosphate (IP3) and diacylglycerol (DAG). IP3 stimulates calcium release from the endoplasmic reticulum, and the resulting increase in intracellular calcium ultimately triggers smooth muscle contraction [154]. However, accumulating evidence suggests that M2 receptors located downstream of the junction also contribute to smooth muscle contraction and may play a more important role than previously appreciated [155]. ACh also acts on presynaptic inhibitory M2 receptors at vagal nerve terminals, where negative feedback limits further ACh release and thereby finely tunes contractile responses. Accordingly, contraction and function of bronchial smooth muscle, gastrointestinal smooth muscle, detrusor muscle, and the pupillary sphincter are all closely related to ACh signaling [156,157,158,159]. It should be noted that carbachol (CCh) is a non-selective cholinergic receptor agonist that is widely used to mimic the effects of ACh. Studies have shown that it increases the proliferation of human detrusor smooth muscle cells (HDSMCs) and induces smooth muscle contraction by activating cholinergic receptors. This effect can be completely blocked by the non-selective muscarinic receptor antagonist atropine, which inhibits smooth muscle contraction and cell proliferation [160]. Similarly, in exocrine glands such as the salivary, lacrimal, and digestive glands, M3 receptor activation stimulates secretion via calcium signaling, supporting enzyme and mucus production for physiological function [161,162]. Research has shown that pilocarpine, as an exogenous mAChR agonist, can significantly stimulate salivary secretion [163]. Likewise, atropine, as an antagonist, can inhibit salivary secretion. Together, these effects represent the core parasympathetic functions of energy conservation, digestion, and absorption.

#### 3.2.3. Immune System

A variety of immune cells, including T cells, B cells, and macrophages, express components of the cholinergic system, including ChAT, AChE, and cholinergic receptors [10]. This non-neuronal cholinergic system (NNCS) plays an important role in immune regulation, particularly in inflammatory and autoimmune diseases [164]. In parallel, cholinergic neurons suppress excessive inflammatory responses through the cholinergic anti-inflammatory pathway, in which the vagus nerve serves a key component [165,166]. Vagus nerve stimulation activates cholinergic signaling in the spleen, leading to ACh releasing and binding to the α7 nAChR on immune cells, thereby suppressing inflammation [167,168]. This mechanism inhibits the production of pro-inflammatory cytokines such as tumor necrosis factor-α (TNF-α), interleukin-1β (IL-1β), and interleukin-6 (IL-6) [169].

In addition, cholinergic signaling modulates immune responses by influencing the differentiation and function of immune cells. Activation of α7 nAChR contributes to T cell development and differentiation and regulates immune responses by modulating ChAT expression and ACh synthesis [170]. Studies have shown that ChAT expression of in T helper 17 (Th17) cells is closely associated with the pathogenesis of experimental autoimmune encephalomyelitis (EAE). Mice with ChAT-deficient Th17 cells are resistant to disease progression and exhibit reduced immune cell infiltration in the brain [171]. Recent evidence indicates that most white blood cells capable of expressing ChAT are B cells, and these cells are associated with macrophage-mediated inflammatory responses [172]. Moreover, α7 nAChR activation inhibits the activity of dendritic cells and macrophages, thereby reducing pro-inflammatory cytokine production [173]. It also promotes the conversion of lipopolysaccharide (LPS)-induced M1 pro-inflammatory microglia into M2 anti-inflammatory microglia [174]. Notably, recent studies have shown that AChE is expressed on the surface of macrophages and may enhance inflammatory responses through interaction with α7 nAChR, suggesting a non-classical role for AChE in inflammatory and providing new insights for future research [175].

## 4. Role of ACh in the Body–Brain Axis

ACh is a highly conserved neurotransmitter that plays a central role in bidirectional communication between the brain and peripheral organs, including the gastrointestinal tract, immune system, cardiovascular system, and liver. Through cholinergic neurons in the CNS, ACh exerts top-down control over peripheral physiology, whereas peripheral cholinergic signaling conveys bodily status to the brain through neural, endocrine, and immune pathways. Together, these mechanisms enable ACh to coordinate diverse physiological processes and maintain systemic homeostasis.

### 4.1. Top-Down Regulation: Central Control of the Periphery

#### 4.1.1. Via the Autonomic Nervous System

**Figure 2:**
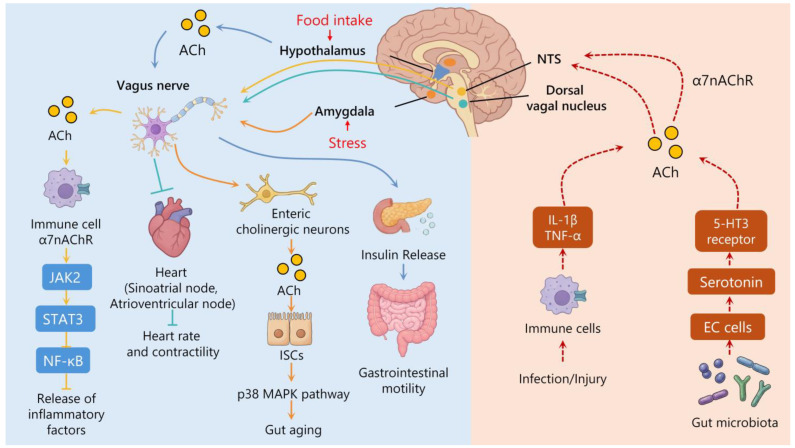
The bidirectional regulatory role of acetylcholine (ACh) in the brain–body communication. ACh mediates top-down central control and bottom-up peripheral feedback via autonomic, neuroendocrine, immune, and microbial pathways. The two different-colored backgrounds in the figure represent the top-down and bottom-up pathways, respectively. With regard to the top-down pathway, brainstem cholinergic signaling enhances vagal activity, lowering heart rate and contractility; hypothalamic ACh promotes insulin release and gastrointestinal motility after food intake. Stress activates the amygdala-dorsal vagal pathway, leading to elevated levels of acetylcholine in the gut. ACh acts on intestinal stem cells (ISCs) and activates the p38 MAPK pathway, thereby inhibiting their self-renewal and accelerating intestinal aging. In terms of the bottom-up pathway, gut microbiota metabolites stimulate enterochromaffin (EC) cells to secrete serotonin (5-HT), activating vagal 5-HT3 receptors and triggering ACh release; ACh signals to the nucleus tractus solitarius (NTS) through α7 nicotinic acetylcholine receptors (α7nAChR). Peripheral infection or injury elicits pro-inflammatory cytokines (TNF-α, IL-1β), detected by vagal afferents and relayed to the NTS. Vagal efferents then drive the cholinergic anti-inflammatory pathway: ACh binds α7nAChR on immune cells, activating JAK2/STAT3 and inhibiting NF-κB to dampen inflammation. Together, these pathways coordinate cardiovascular function, feeding behavior, gut homeostasis, stress responses, and immune regulation.

The autonomic nervous system integrates sympathetic and parasympathetic outputs to regulate organ function, and ACh is a key mediator in this process. Central cholinergic signaling enhances vagal activity via muscarinic receptors in the brainstem, including the nucleus of the solitary tract and dorsal motor nucleus of the vagus, thereby reducing heart rate and contractility through direct effects on the sinoatrial and atrioventricular nodes (Figure 2) [176,177]. In contrast, sympathetic activation increases cardiac output through norepinephrine and epinephrine release [178]. Rather than acting as a simple antagonist to sympathetic signaling, central ACh contributes to dynamic autonomic balance. During exercise or stress, a “cholinergic brake” may transiently limit excessive sympathetic activation and stabilize cardiovascular function [179].

ACh also participates in central regulation of gastrointestinal physiology. Chronic stress activates the amygdala–dorsal vagal nucleus pathway, increasing vagal outflow and stimulating ACh release from enteric cholinergic neurons. In the gut, ACh acts on M3 receptors on intestinal stem cells (ISCs) and activates the p38 MAPK pathway, impairing stem cell self-renewal and accelerating intestinal aging (Figure 2) [180]. In addition, hypothalamic cholinergic signaling contributes to feeding-related autonomic regulation. After food intake, increased ACh enhances vagal tone, promotes insulin secretion and gastrointestinal motility, and indirectly suppresses sympathetic activity, thereby reducing heart rate and blood pressure and facilitating energy storage and nutrient absorption (Figure 2) [181,182].

#### 4.1.2. Via the Neuroendocrine Axis

In addition to neural pathways, ACh can regulate peripheral function through the neuroendocrine system. The hypothalamic–pituitary–adrenal (HPA) axis is the principal endocrine pathway for stress responses. In this axis, hypothalamic corticotropin-releasing hormone (CRH) stimulates pituitary adrenocorticotropic hormone (ACTH) release, which in turn induces glucocorticoid secretion from the adrenal cortex [183]. nAChRs are expressed in the paraventricular nucleus (PVN) of the hypothalamus, and cholinergic afferents may innervate PVN-adjacent neurons that project to CRH neurons [184]. ACh activation in the PVN promotes the release of CRH and arginine vasopressin (AVP), thereby stimulating the HPA axis [185]. In rats, ACh preferentially increases CRH secretion, suggesting stimulus-specific regulation of the HPA axis by cholinergic signaling [186]. Inflammatory cytokines can also activate the HPA axis by stimulating hypothalamic CRH release, linking cholinergic anti-inflammatory signaling to endocrine control of stress responses [187].

### 4.2. Bottom-Up Regulation: Peripheral Influence on the Brain

#### 4.2.1. Via Gut–Brain Axis

The gut–brain axis is a bidirectional communication network linking the enteric nervous system (ENS) and the CNS through neural, endocrine, immune, and microbial signals [188]. Often referred to as the “second brain,” the ENS contains ganglia in the submucosal and myenteric plexuses that are connected by internodal chains to support rapid signal transmission along the intestinal tract [189]. Cholinergic neurons are abundant in both plexuses, and peripheral choline acetyltransferase (pChAT) serves as a marker of ENS cholinergic neurons throughout the gastrointestinal tract from the esophagus to the rectum [190,191,192].

Gut microbiota-derived metabolites also influence cholinergic signaling. Short-chain fatty acids (SCFAs), including acetate, propionate, and butyrate, stimulate enterochromaffin (EC) cells to release serotonin (5-HT) [193]. Serotonin then activates vagal afferents via 5-HT3 receptors, triggering ACh release [194]. ACh further transmits information to the nucleus tractus solitarius (NTS) via α7 nicotinic receptors on vagal terminals (Figure 2) [195,196]. The NTS then relays this information to brain regions such as the locus coeruleus, hypothalamus, amygdala, and prefrontal cortex, thereby influencing mood, appetite, cognition, and stress responses [197]. Moreover, the gut microbiota is closely associated with the development of Alzheimer’s disease (AD) [198]. Studies have shown that dysbiosis of the gut microbiota can activate central immune responses by disrupting the intestinal mucosal barrier and promoting the release of inflammatory cytokines. This subsequently exacerbates the deposition of β-amyloid (Aβ) and neuroinflammation in the brain, ultimately accelerating the pathological progression of AD [199]. This suggests a potential link between gut microbiota dysbiosis in AD patients and the cholinergic anti-inflammatory pathway, which may represent an essential therapeutic target for future AD treatments.

In addition, the relationship between botulinum toxin (BoNT) and the gut–brain axis is becoming increasingly clear. BoNT is a potent neurotoxin produced by Clostridium botulinum. It consists of a 50 kDa light chain and a 100 kDa heavy chain [200]. BoNT inhibits the release of ACh by binding to soluble N-ethylmaleimide-sensitive factor attachment protein receptors (SNAREs) on the presynaptic membrane, thereby preventing the fusion of ACh-containing vesicles with the nerve terminal membrane [201]. This process can lead to slowed intestinal motility and reduced secretion, thereby affecting gastrointestinal motility [202]. This mechanism is clinically applied in the treatment of various gastrointestinal diseases and is currently a hot topic of research.

#### 4.2.2. Via the Inflammatory Response

ACh plays a bidirectional regulatory role in the modulation of immunity and inflammation. It can not only suppress excessive inflammatory responses but also promote defensive inflammatory responses and the migration of immune cells. Peripheral infection or tissue injury triggers the release of pro-inflammatory cytokines such as TNF-α and IL-1β [203]. These inflammatory signals are detected by vagal afferent fibers, which relay information to the NTS in the brainstem [204]. As a central hub in the CNS for integrating visceral sensory information, the NTS processes immune-related signals and engages the cholinergic anti-inflammatory pathway via vagal efferents to suppress excessive inflammation [205]. In this pathway, ACh released from vagal terminals binds to α7nAChR on macrophages and other immune cells [206]. This activates JAK2/STAT3 signaling and inhibits NF-κB-dependent cytokine transcription, thereby reducing pro-inflammatory mediator production (Figure 2) [207]. α7nAChR also limits monocyte-derived macrophage migration during acute inflammation [208]. This rapid neuroimmune mechanism functions independently of the HPA axis and represents an important target for therapeutic intervention. Additionally, ACh can also play a role in promoting inflammatory responses. In response to infection, T cells upregulate ChAT expression and release ACh, which acts on the M3 muscarinic receptors of vascular endothelial cells to induce the production of nitric oxide (NO). NO causes vasodilation, thereby facilitating the migration of immune cells to infected tissues or sites of inflammation [209]. This is crucial for effectively controlling the pathological process of infection.

## 5. Summary

ACh is a highly conserved neurotransmitter that integrates central and peripheral physiology through precisely coordinated signaling pathways. In the CNS, cholinergic circuits regulate cognition, learning and memory, attention, arousal, reward, and decision-making. In the PNS, ACh mediates neuromuscular transmission and autonomic regulation, thereby controlling both voluntary and involuntary physiological processes. Importantly, ACh also serves as a key mediator of the brain–body axis, linking top-down autonomic and neuroendocrine control with bottom-up signals from the gut and immune system. Collectively, these functions position ACh as a fundamental regulator of systemic homeostasis and a promising therapeutic target for neurodegenerative, psychiatric, cardiovascular, and inflammatory diseases (Figure 2). Future studies should further clarify the spatiotemporal specificity of cholinergic signaling, its cell-type-dependent actions, and its interactions with other neuromodulatory and immune pathways, which may facilitate the development of more precise cholinergic-based interventions.

In the past, treatments targeting the cholinergic system have often had limited efficacy, primarily due to two main limitations. On one hand, they only alleviate symptoms and fail to halt neurodegenerative changes. Acetylcholinesterase inhibitors (AChEIs) improve neural signal transmission by increasing acetylcholine levels in the synaptic cleft, thereby alleviating cognitive and behavioral symptoms [210]. However, they do not address the underlying pathology of AD, meaning that AChEIs cannot halt disease progression, and their effects are often short-lived. On the other hand, side effects can result from a lack of receptor subtype selectivity. Most current AChEIs non-selectively increase acetylcholine levels throughout the brain and even in peripheral tissues. This can lead to unintended side effects, such as gastrointestinal and cardiovascular adverse reactions like nausea, vomiting, diarrhea, and bradycardia, thereby compromising the efficacy of drug therapy [211].

Therefore, therapeutic strategies targeting the cholinergic system will focus on more selective and multi-target approaches to overcome the limitations of current treatments in the near future. M1 muscarinic receptors play a critical role in cognitive function, and their dysfunction is associated with neurocognitive disorders such as Alzheimer’s disease (AD) [212]. Selective M1 muscarinic receptor positive allosteric modulators (PAMs) can enhance the action of endogenous acetylcholine at M1 receptors without causing widespread muscarinic receptor desensitization or peripheral side effects. Current research is focused on developing M1 PAMs for the treatment of neurocognitive disorders by targeting the allosteric sites of M1 muscarinic receptors, with the aim of improving conditions such as AD and schizophrenia [213]. Furthermore, the development of multi-target directed ligands (MTDLs) capable of acting on multiple disease-causing targets simultaneously represents the cutting edge of current research. AD is a multifactorial disease, and drugs targeting a single pathway often have limited efficacy. Licochalcone A not only acts as an acetylcholinesterase inhibitor but also exerts neuroprotective effects against Alzheimer’s disease by inhibiting tau protein phosphorylation, reducing Aβ plaque deposition, and alleviating neuroinflammation [214].

## Data Availability

No new data were created or analyzed in this study. Data sharing is not applicable to this article.
